# Challenges for the implementation of next generation sequencing-based expanded carrier screening: Lessons learned from the ciliopathies

**DOI:** 10.1038/s41431-022-01267-8

**Published:** 2022-12-23

**Authors:** Ella Vintschger, Dennis Kraemer, Pascal Joset, Anselm H. C. Horn, Anita Rauch, Heinrich Sticht, Ruxandra Bachmann-Gagescu

**Affiliations:** 1 Institute of Medical Genetics, University of Zurich, 8952 Schlieren, Switzerland; 2 Institute of Biochemistry, Friedrich-Alexander-Universität Erlangen-Nürnberg (FAU), 91054 Erlangen, Germany; 3 Praeclare Clinical Research Priority Program of the Medical Faculty, University of Zurich, Zurich, Switzerland; 4 Department of Molecular Life Sciences, University of Zurich, 8057 Zurich, Switzerland; 5 Present Address: Institute of Medical Genetics and Pathology, University Hospital Basel, 4031 Basel, Switzerland

**Keywords:** Risk factors, Pregnancy outcome

## Abstract

Next generation sequencing (NGS) can detect carrier status for rare recessive disorders, informing couples about their reproductive risk. The recent ACMG recommendations support offering NGS-based carrier screening (NGS-CS) in an ethnic and population-neutral manner for all genes that have a carrier frequency >1/200 (based on GnomAD). To evaluate current challenges for NGS-CS, we focused on the ciliopathies, a well-studied group of rare recessive disorders. We analyzed 118 ciliopathy genes by whole exome sequencing in ~400 healthy local individuals and ~1000 individuals from the UK1958-birth cohort. We found 20% of healthy individuals (1% of couples) to be carriers of reportable variants in a ciliopathy gene, while 50% (4% of couples) carry variants of uncertain significance (VUS). This large proportion of VUS is partly explained by the limited utility of the ACMG/AMP variant-interpretation criteria in healthy individuals, where phenotypic match or segregation criteria cannot be used. Most missense variants are thus classified as VUS and not reported, which reduces the negative predictive value of the screening test. We show how gene-specific variation patterns and structural protein information can help prioritize variants most likely to be disease-causing, for (future) functional assays. Even when considering only strictly pathogenic variants, the observed carrier frequency is substantially higher than expected based on estimated disease prevalence, challenging the 1/200 carrier frequency cut-off proposed for choice of genes to screen. Given the challenges linked to variant interpretation in healthy individuals and the uncertainties about true carrier frequencies, genetic counseling must clearly disclose these limitations of NGS-CS.

## Introduction

Next generation sequencing (NGS) has revolutionized medical genetics and contributed to the identification of thousands of genes associated with Mendelian diseases, providing a molecular diagnosis to many affected individuals. In parallel, this technology offers unprecedented possibilities for preventing disease, in particular in the context of preconception carrier screening. Indeed, many diagnostic laboratories offer NGS-based carrier screening (NGS-CS, or expanded carrier screening) [1], with the promise of allowing couples to make informed reproductive decisions. The range of commercial offers includes gene panels of various sizes (up to >500 different genes) where all identified sequence variants are analyzed (rather than limiting to known disease alleles). Moreover, rather than focusing on selected populations at increased risk for a few recessive disorders, recent publications have demonstrated an increased effectiveness for comprehensive pan-ethnic NGS-CS [2]. Based on such findings and on the improved technical performances and reduced cost of NGS, the latest ACMG recommendations support offering NGS-CS for recessive and X-linked conditions in an ethnic and population-neutral manner to all women/couples for all genes that have a carrier frequency >1/200 based on GnomAD data [3]. However, despite the well-established technical reliability of NGS for confidently identifying sequence variants [4, 5], assessing the clinical validity of results remains challenging [6], especially for populations less intensively sequenced [7]. Indeed, the clinical validity of a screening test depends on high positive and negative predictive values [8], as discussed in the statement paper of the ESHG on expanded carrier screening which also recommends reporting only variants that are clearly pathogenic [9]. Following this recommendation, the majority of laboratories offering NGS-CS report only variants considered pathogenic (*P*) according to ACMG/AMP criteria [10]. Unfortunately, application of these guidelines in the context of carrier screening for recessive disorders is challenging because several criteria are not applicable in healthy individuals without any phenotype or family history [10]. Many variants are therefore typically classified as variants of unknown significance (VUS, ACMG/AMP class 3) which should not be reported, but whose existence will decrease the negative predictive value, since some VUS may be disease alleles. On the other hand, some variants previously classified as pathogenic without sufficient evidence may turn out not to be disease-causing, leading to false positive test results and reduced positive predictive value.

In order to determine to which extent uncertainties in variant interpretation affect NGS-CS in the current state of knowledge, we focused on the ciliopathies, a group of recessive Mendelian disorders [11, 12]. Ciliopathies are defined by their association to a quasi-ubiquitous cellular organelle called the primary cilium, which acts as a cellular antenna for signal transmission [13]. Ciliopathies are characterized by clinical pleiotropy, with a recurring set of phenotypes (central nervous system anomalies/dysfunction, retinal degeneration, cystic kidneys, polydactyly, bone anomalies) with important phenotypic variability. Ciliopathies typically manifest in childhood and are sufficiently severe to justify prenatal testing, in line with the current recommendations for carrier screening [9, 14]. Indeed, a subset of ciliopathy genes are included in the ACMG-recommended “Tier 3” genes, for which carrier screening should be offered to all individuals. Ciliopathies are further characterized by prominent allelic and genetic heterogeneity. Therefore, the large number of ciliopathy genes increases the likelihood of identifying sequence alterations in one of them in any given individual. As an asset in the context of NGS-CS, ciliopathies are intensively studied, so that the large body of literature describing the genetic makeup of ciliopathy cohorts and our understanding of ciliary biology can be leveraged to improve variant interpretation.

In this work, we analyzed 118 ciliopathy genes by whole exome sequencing (WES) in ~400 healthy individuals and confirmed the results in ~1000 individuals from the UK1958 birth cohort. We find that 11–20% carry at least one pathogenic variant in a ciliopathy gene, while ~50% harbor one or more rare “strong” VUS. For 4% of couples the risk of having offspring affected by a ciliopathy cannot be excluded. Even when focusing only on pathogenic and loss-of-function variants, we find carrier frequencies that are substantially higher than expected based on estimated disease prevalence, demonstrating that the actual carrier frequency remains unclear for most ciliopathy genes and that the proposed 1/200 carrier frequency cut-off merits re-assessment. Our findings illustrate the still very high proportion of VUS and the current limitations of variant databases. Finally, we show that incorporating additional insights gained from structural protein information and gene-specific knowledge from the literature may assist in prioritizing variants.

## Methods

### Study cohorts

The primary study cohort consisted of 395 healthy individuals (demographic information in Supplementary Table S1). The comparison cohort was comprised of 999 participants from the UK1958 National Child Development Study (https://ega-archive.org/datasets/EGAD00001001021). All study participants provided written informed consent and the research was approved by the cantonal ethics committee of Zurich, references StV 11/09 and BASEC-Nr.2019-00016.

### Whole-exome sequencing (WES)

For the primary cohort, WES was performed on DNA extracted from peripheral blood lymphocytes using Agilent (Santa Clara, CA, USA) SureSelect XT Clinical Research Exome Kit (V5) or Human All Exon (V6), followed by bi-directional paired-end sequencing of 125 base pairs (bps) on a HiSeq2500 platform (Illumina, San Diego, California, USA). Alignment to the hg19 reference genome was performed using NextGENe software (Softgenetics, State College, Pennsylvania, USA). Cut-off values for alternate read fraction and minimum coverage were 16% of supporting reads and 20× read depth, respectively. WES data with <85% of the exome covered at <20× read depth were considered as insufficient quality. Variant calling was restricted to the selected 118 ciliopathy target genes (Supplementary Table S2). Per sample WES raw sequencing data from the UK1958 Birth cohort (https://ega-archive.org/studies/EGAS00001000971) were realigned using NextGen software. The same quality criteria and filters were applied to this dataset. Segregation analysis for the local cohort was performed using WES data from the offspring: if both or neither of the variants found in a parent were present in the child, then the variants were in *cis* in the parent.

### Variant filtering

Python 3.7.4 was used for programming. Manual pre-processing was performed to exclude calling artefacts as described in the Supplementary methods. To retain only high confidence variants (“true calls”), we trained a linear support vector machine (Supplementary methods, Supplementary Fig. S1). Pathogenic variants were determined according to ACMG/AMP criteria: PVS1 (loss-of-function alleles) + PM2 (GnomAD allele frequency (MAF) ≤ 0.1%) AND/OR previously classified as pathogenic (*P*) in Clinvar /disease associated (*DM*) in HGMD. Novel very rare (MAF ≤ 0.1%) loss-of-function alleles (frameshift, stop gain and canonical splice site variants) were retained in a second step. Finally, variants that were very rare (MAF ≤ 0.1%) and for which a majority of prediction algorithms predicted a deleterious effect were retained as “strong” VUS. The following standard databases/algorithms were used (Supplementary methods): GnomAD, ClinVar, HGMD professional, CADD, M-Cap, SIFT, Polyphen2, LRT, Mutation Assessor, Mutation Taster, FATHMM, PROVEAN.

## Results

### Cohorts and NGS sequencing

We performed WES on 395 healthy individuals who were the parents (176 couples, of which 21 were consanguineous, and 43 single parents, Supplementary Table S1) of children referred to the pediatric genetics clinic for a variety of indications (developmental delay/intellectual disability, malformations, dysmorphisms etc). None of the children had a clinically suspected ciliopathy disorder. To rule out a selection bias, we performed the same analysis on WES data from 999 individuals from the UK1958 birth cohort. Average coverage was 286.9 reads/basepair for the local cohort and 57.2 reads/basepair for the UK1958 cohort (Supplementary Fig. S2).

### Variants in 118 recessive ciliopathy genes

**Fig. 1:**
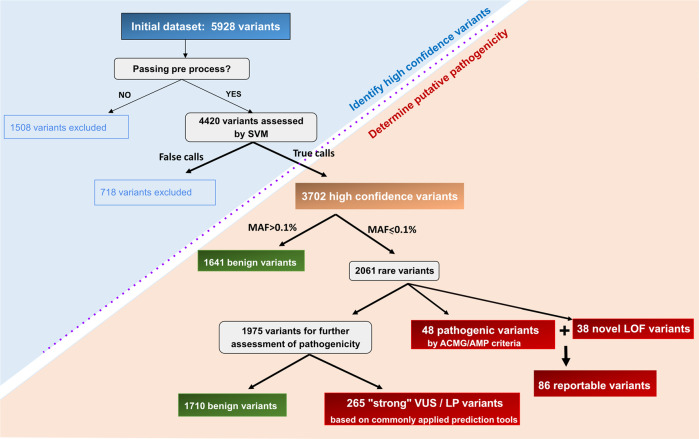
Variant interpretation pipeline. Filtering pipeline for all variants in 118 ciliopathy-associated genes identified by whole exome sequencing in 395 healthy controls. The blue area indicates the filtering of variants to retain only high quality variants (“true calls”). *SVM* stands for support vector machine. The pink area describes filtering steps to determine pathogenicity of very rare variants (MAF ≤ 0.1%). Pathogenic variants are determined according to ACMG/AMP criteria. “Strong VUS” were determined as variants that are possibly damaging based on prediction tools: CADD > 20, M-CAP > 0.025, and 4 of the remaining 7 tools (SIFT, Polyphen2, FATHHM, MutationTaster, MutationAssessor, PROVEAN, LRT) predicting deleteriousness and/or previous classification in HGMD as *DM* or in ClinVar as *LP* with limited supporting information disclosed. For details please see methods.

We filtered the WES data for variants in 118 genes associated with a range of ciliopathies (Supplementary Table S2), selected based on OMIM, literature searches and specialized reference websites (http://www.syscilia.org/goldstandard.shtml) and identified 5928 variants. After manual pre-processing excluding variants that were calling artefacts (Supplementary methods) 4420 variants were retained. By training a support vector machine (Supplementary methods, Supplementary Fig. S1) we excluded 718 variants as false calls, leaving 3702 high confidence variants for further analysis (Fig. 1). The average number of high-confidence variants was 9.32 per individual (range 1–24 variants/individual) (Supplementary Table S3). We performed the same analysis for the UK1958 cohort and found similar results, identifying a total of 7377 high-confidence variants (average 7.38 variants/individual, range 1–19 variants/individual; Supplementary Table S3). The slightly lower numbers in the UK1958 cohort may be explained by the lower WES coverage yielding fewer high confidence variants.

### Reportable pathogenic variants following ACMG/AMP criteria

The next step focused on retaining pathogenic variants according to ACMG/AMP criteria [10]. These included all very rare variants (PM2 - minor allele frequency MAF ≤ 0.1%) that were either predicted to result in loss-of-function (LOF: nonsense, frameshift, canonical splice site – PVS1) AND were previously reported and classified as pathogenic (*P*) in ClinVar AND/OR disease causing in HGMD (DM) OR rare missense variants that were classified as *P* in ClinVar AND *DM* in HGMD with sufficient supporting information. We also retained two known recurring alleles in ciliopathy genes which have allele frequencies >0.1% in GnomAD: *BBS1* (NM_024649.4) c.1169 T > G, p.M309R [15] and *KIAA0586* (NM_001244189.1) c.428delG, p.R143Kfs*4 [16, 17]. Applying these criteria, 48 pathogenic variants were found in 43 individuals, of which 26 were predicted to be LOF, and 22 missense, in-frame indels or non-coding variants (Table 1, Fig. 1, Supplementary Table S4). The carrier frequency for pathogenic variants in one of 118 ciliopathy genes in this cohort of healthy individuals lies at ~11% (43/395). One individual carried 2 variants in the same gene, but segregation analysis revealed that the two variants were *in cis*. For one consanguineous couple, both partners were carriers of a pathogenic missense variant in *DYNC2H1*, where the offspring had not inherited both variant alleles (Supplementary Table S5). Interestingly, for several genes, we observed much higher carrier frequencies than expected based on estimated disease prevalence. The well-known recurrent pathogenic variants *KIAA0586* (NM_001244189.1) c.428delG, p.R143Kfs*4 or *BBS1* (NM_024649.4) c.1169 T > G, p.M309R variants, whose disease-association is supported by a large body of literature and/or functional studies [15–18] were each identified in three unrelated individuals in our cohort (0.75%). Moreover, for *CEP290*, we identified previously described pathogenic LOF variants in four individuals (0.75%).

**Table 1:** Number of variants identified in our local cohort: reportable variants (light grey) and “strong” VUS (darker grey).

	Reportable variants (*pathogenic* by ACMG/AMP criteria)		“Strong” VUS * or *likely pathogenic* by ACMG/AMP criteria	
Total variants	86		265	
	**Variants** ***P*** **ClinVar**	**48**	Variants *LP* ClinVar	4
	LOF/truncating	26	Variants *DM* HGMD	40
	Missense/noncoding/in-frame	22	LOF variants VUS ClinVar	3
	**Novel** **predicted LOF/truncating variants**	**38**	Missense/noncoding/in-frame (VUS, conflicting or absent ClinVar; DM? or absent HGMD)	218
**Individuals carrying variants** (**%**)	**80 (20%)**		**197 (50%)**	
**N variants/individual (range)**	0–2		0–5	
**Avg variants/individual**	0.23		0.66	
**Couples at risk (%)**	2 /177 (1%)		7/177 (4%)	

*“Strong” VUS were defined as very rare (MAF ≤ 0.1%) *AND* [HGMD DM or DM?] *OR* [ClinVar LP] with limited supporting information provided *OR* [CADD ≥ 20 *AND* M-Cap≥ 0.025 AND 4 of the 7 remaining prediction tools (SIFT, Polyphen2, LRT, Mutation Assessor, Mutation Taster, FATHMM, PROVEAN) with deleterious/pathogenic classification].

*DM* disease associated, *HGMD* human gene mutation database, *LOF* loss of function, *N* number*, P* pathogenic, *LP* likely pathogenic, *VUS* Variant of uncertain significance.

### Novel predicted loss-of-function alleles

The advantage of performing WES rather than using a pre-set variant detection panel, is that novel variants can also be identified. We detected in addition 38 predicted LOF variants that have not been previously classified in ClinVar or HGMD in 36 individuals. These variants should be formally classified as *likely pathogenic* based on ACMG/AMP criteria PVS1 (truncating variant) and PM2 (allele frequency-very rare). Since loss-of-function is the most common genetic disease mechanism in ciliopathies for all the genes analyzed, the majority of these variants will be disease-causing and it remains open for discussion, whether they should be reported in the context of carrier screening. According to Tavtigian et al., who analyzed the ACMG/AMP criteria in a bayesian framework, the “likely pathogenic rule” PVS1 + one moderate evidence of pathogenicity (i.e. rarity PM2) yielded a posterior probability of 0.994 for pathogenicity, similar to other “pathogenic rules” in this classification [19]. Following this rationale, novel very rare truncating variants in a known ciliopathy gene should probably be reported even in the context of carrier screening. If such variants are included, this raises the overall carrier frequency for ciliopathies in our cohort to 20% (80/395 individuals). For some genes such as *KIAA0586, CEP290* or *ALMS1*, the carrier frequency would rise to 1.7 respectively 1.3%, which is substantially higher than expected based on disease prevalence which is estimated at between ~1:50’000 and 1:1’000’000 [20–23]. Including novel LOF variants, one additional consanguineous couple in our cohort would be at risk for having an affected child (total 2/21 consanguineous couples = 10% or 2/176 couples = 1%).

To rule out a selection bias in our local cohort, we applied the same filtering steps to the UK1958 cohort and found results within the same order of magnitude: 8% of the individuals in the UK1958 cohort were carriers for a *P* variant in a ciliopathy gene, and up to 15% when including very rare novel LOF variants (Supplementary Table S3). The slightly higher proportion of variants in the local cohort compared to the UK1958 cohort may be explained by the poorer sequencing coverage in the latter, which led to higher exclusion rates of variants of poor quality (Supplementary Fig. S2).

### Variants of uncertain significance (VUS)

Ideally, a screening test should have a high positive predictive value, identifying (almost) all true carriers, but also a high negative predictive value, where a negative test excludes with sufficient confidence that someone is a carrier. While the technical reliability of NGS is now excellent, and all variants that are present are likely to be detected, the question focuses more on how many variants, which cannot be certainly classified as pathogenic or benign, each individual carries.

To quantify this problem, we further analyzed our WES data for missense variants which we deemed to be “strong” VUS. As VUS, these variants are not to be reported in the context of carrier screening – the purpose of this analysis was rather to define the amount of variants that might contain disease alleles, which would decrease the negative predictive value of the test. Such variants would be retained by automatic filtering pipelines based on previous classifications in ClinVar or HGMD or based on allele frequency (MAF ≤ 0.1%) AND strict combination of deleteriousness prediction from multiple algorithms (criteria chosen: CADD > 20 AND MCAP > 0.025 AND ≥ 4 of remaining 7 algorithms predicting a deleterious effect). The reasoning behind this selection of criteria is that the interpretations often differ between prediction tools. Indeed, analyzing the correlation between the different tools using the numerical scores they provide, we found the Pearson correlation coefficient to range between ~−0.1 and ~0.7 (Supplementary Fig. S3). Variants retained in this part of the analysis therefore required deleteriousness prediction by 6/9 algorithms (CADD and MCAP being accounted for separately since they in part rely on the other tools). Four variants classified as *LP* in ClinVar with limited supporting information were considered as VUS.

**Fig. 2:**
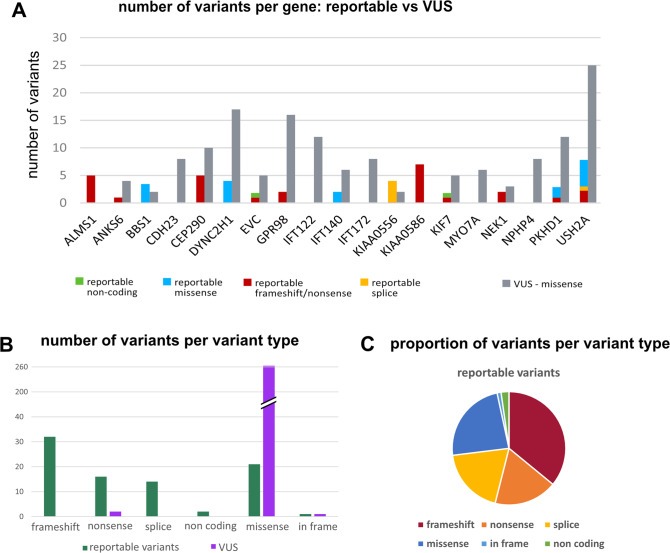
Comparison of variants per gene: reportable vs “strong” VUS. **A** Number of variants per ciliopathy gene that are reportable (left bar for each gene: pathogenic or novel LOF) vs variants of uncertain significance VUS (right grey bar for each gene). Only genes where 5 or more individuals in this cohort showed variants are depicted. Variant types are color-coded in each bar: *blue* indicates missense (or in-frame indels), *red* nonsense/frameshift, *yellow* canonical splice site, *green* non-coding, and *grey* “strong” VUS. Note the different distribution of variant types per gene (truncating vs missense) and the increase in variants for some genes, but not for others between reportable variants and VUS (increase mostly for *USH2A, GPR98/ADGRV1* and *DYNC2H1*). **B** Number of variants in ciliopathy genes shown per variant type for reportable variants (green bars) and unclear variants VUS (purple bars): as expected, the vast majority of VUS are missense variants. **C** Proportion of variant types (color-coded as above in (**A**) for the reportable variants (previously classified pathogenic and novel loss-of-function variants).

This analysis led to the detection of 262 additional missense/in-frame variants. Interestingly, the number of missense VUS differed between genes, with the largest increase for *USH2A, ADGRV1/GPR98 and DYNC2H1* (Fig. 2). 23 missense variants recurred in more than one individual (Supplementary Table S6), such that there were a total of 239 unique missense variants. 197/395 (50%) individuals carried between 1–5 “strong” VUS, of which an unknown proportion may be disease-causing alleles, in the 118 analyzed ciliopathy genes. Six individuals had two VUS in the same gene but segregation analysis in their offspring demonstrated that they were *in cis* in all but one case. VUS were found in both partners in 7 non-consanguineous couples (7/176 = 4%). The offspring inherited both variants in two cases: one child was homozygous for a missense variant in *IFT81* and one was compound heterozygous for two *USH2A* missense variants. Neither child displayed phenotypes consistent with the associated disorders, indicating that at least some of these variants are not disease-causing. Similar results were found in the UK1958 cohort, with 45% of individuals carrying between 1 and 5 VUS (Supplementary Table S3).

Among the missense variants retained as “strong” VUS, 42 had been previously classified as *DM* in HGMD and sometimes as *P*/*LP* by some providers in ClinVar (but with conflicting interpretations of pathogenicity, mostly VUS vs *P/LP*). We judged these previous classifications to be insufficient to definitely retain these variants as pathogenic, based for example on the fact that 20 *DM* variants in HGMD were benign in ClinVar (Supplementary Fig. S4). This underscores the limitations of these commonly used databases. One could expect their performance to increase with time as the global sequencing experience increases. To analyze this, we compared the ClinVar classification for 400 missense variants from our dataset between 2018 and 2020. While we did observe an increase in the proportion of missense variants that had a ClinVar entry (from 52% to 60%), it was mostly variants with conflicting interpretations of pathogenicity that increased (Supplementary Fig. S4 and Table S4). One variant changed from pathogenic to benign. These data illustrate how difficult it remains to determine variant pathogenicity, in particular when several ACMG/AMP criteria cannot be used for classification, as is the case for healthy individuals.

Taken together, these results show that ~50% of this healthy population carries a “strong” VUS in a ciliopathy gene and that a reproductive risk for a ciliopathy *remains possible* in 4% of couples. While it is not advisable to report variants that are not unequivocally pathogenic, not reporting all these VUS will decrease the negative predictive value of the screening test. On the other hand, the higher-than-expected carrier frequencies even for LOF alleles and the database limitations suggest that determining what is a “clearly” pathogenic variant remains a moving target.

### Approaches for further evaluation of VUS in healthy individuals

The crux of the problem lies in the classification of the variants. Indeed, the large majority of missense variants identified in healthy individuals will not be disease alleles, foremost because ciliopathies are rare disorders. To identify which VUS are most likely disease-causing, we sought to leverage existing knowledge on ciliopathies by comparing the identified variants with known disease alleles from patient cohorts.

**Fig. 3:**
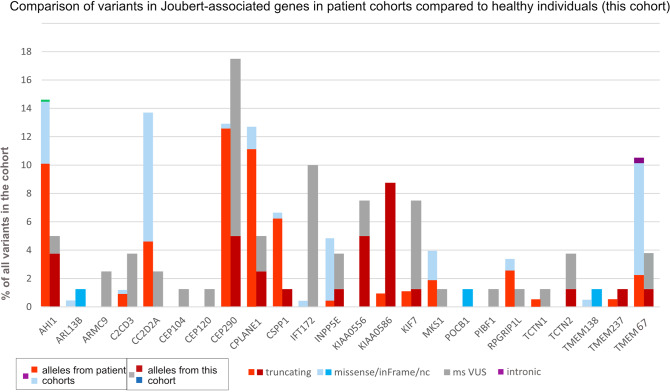
Comparison of variants per gene and variant type in Joubert syndrome-associated genes in a patient cohort compared to healthy individuals (this cohort). Bar graph showing all Joubert-associated genes in which variants discussed here (reportable or “strong” VUS) were found in this healthy cohort. The percentage of variants in each gene (over the total number of variants in each cohort) is shown for both cohorts: for each gene, the left bar, consisting of light red (truncating) and light blue (missense/nc/inframe variants) variants, shows results from a large Joubert-patient cohort [25] as a comparison, while the right bar (darker red for truncating, darker blue for reportable missense/nc/inframe and grey for “strong” VUS) shows the results from the current study in healthy individuals. Note the substantial differences for instance for *CEP290*, where affected individuals have mostly truncating variants, while healthy individuals in this cohort have predominantly missense variants, or for *IFT172*, where pathogenic alleles rarely cause JBTS while many “strong” VUS were identified in this healthy cohort. Such a difference is not present for all genes (for example variants in *INPP5E* show a similar distribution between affected and healthy individuals).

We focused on the well-studied Joubert syndrome (JBTS) [23, 24], which is characterized by a specific hindbrain malformation and which can be caused by bi-allelic mutations in one of ~40 different ciliopathy genes [25, 26]. The five genes most commonly mutated in individuals with JBTS are *AHI1, CC2D2A, CEP290, C5orf42/CPLANE1* and *TMEM67* and the disease alleles are predominantly truncating/LOF variants [25, 27]. In comparison, variants in these genes identified in our healthy local population were mostly missense variants, most abundant in *CEP290, KIF7* and *IFT172*. Since *IFT172* and *KIF7* are only uncommonly mutated in patients, the true carrier frequency for disease alleles for these genes is expected to be very low, suggesting that most *IFT172* or *KIF7* variants identified in our healthy cohort are benign. For *CEP290 or KIF7*, the majority of variants in our cohort were missense variants, while virtually all described disease alleles in patients are LOF [25, 28–31] (Fig. 3). For other genes such as *INPP5E* however, the majority of known disease alleles are missense variants [25, 32]. The missense variants identified in our cohort in *INPP5E* are therefore more likely to represent disease alleles than those in *CEP290* or *KIF7*. Taken together, these data illustrate how knowledge of the mutation pattern in patients can help to interpret pathogenicity of novel variants. To capture this knowledge, we determined a simple “missense score” for each gene based on the “clinical statistics” table in *varsome* [33], by calculating the ratio of missense *P/LP* variants over all *P/LP* variants (Supplementary Table S7).

### Incorporating structural protein information for further variant prioritization

**Fig. 4:**
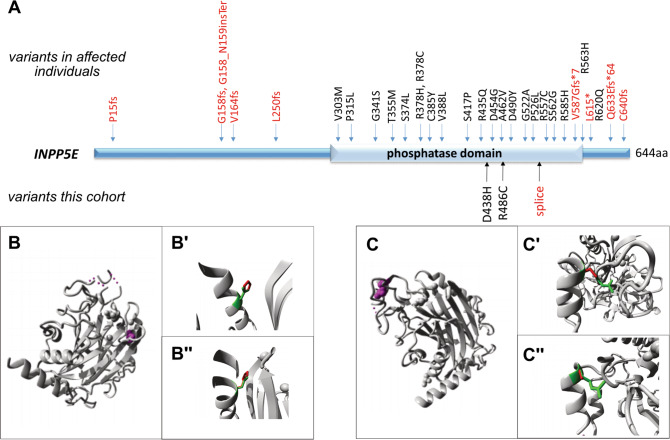
Scrutiny of individual variants: example for *INPP5E* variants. **A** Schematic of INPP5E protein showing the phosphatase domain. On top are displayed missense variants described in individuals with Joubert syndrome from the literature, at the bottom are the variants identified in the healthy individuals in our cohort. Note the clustering of missense variants in the phosphatase domain in affected individuals. The two missense variants identified in our cohort localize just next to previously described disease alleles. **B**–**C”** Protein modeling using HOPE software [34] for the two *INPP5E* missense variants identified in the healthy individuals in our cohort. **B**, **C** Overview of the protein in ribbon-presentation. The protein is colored grey, the mutated residue is colored magenta. **B’**–**B”, C’**–**C”** Close-ups of the mutations. The protein is colored in grey, the side chains of wild-type and mutant residues are colored green and red, respectively. **B D438H**: The HOPE report indicates that the wild-type Aspartic acid residue forms a hydrogen bond with Threonines at positions 395 and 426. The size difference between wild-type and mutant residues makes that the new residue is not in the correct position to make the same hydrogen bond as the original wild-type residue did. In addition, the wild-type residue forms a salt bridge with Arginines at position 396, 435, and 441. The difference in charge will disturb the ionic interaction made by the original, wild-type residue. **C R486C**: The HOPE report indicates that the wild-type Arginine residue forms hydrogen bonds with Aspartic Acid at position 544, Arginine at position 550 and Tyrosine at position 543. The size difference between wild-type and mutant residues makes that the new residue is not in the correct position to make the same hydrogen bonds as the original wild-type residue did. The difference in hydrophobicity will affect hydrogen bond formation. In addition, the wild-type residue forms a salt bridge with Aspartic Acid at positions 490, 537 and 544. The difference in charge will disturb the ionic interaction made by the original, wild-type residue.

We next sought to include structural protein information where available. We performed in silico protein modeling using HOPE [34] for those protein regions for which structural data was available. As an illustration, the HOPE report for the missense variants identified in *INPP5E*, which are located in the phosphatase domain, suggests that they may disrupt protein function (Fig. 4). To incorporate structural protein information into our pipeline in an automated manner, we turned to VIPUR, a Rosetta-based scoring system that relies on structural information in addition to classical sequence-based predictions [35]. VIPUR allots each variant a score between 0 and 1, where a score > 0.5 suggests deleteriousness, and a score > 0.7 strongly suggests deleteriousness. Of 249 unique missense VUS from our dataset, VIPUR called 102 deleterious, 78 neutral and was unable to classify 66 due to lacking structural information. Integrating the VIPUR score and the “missense score” (ms-score) with the previously described criteria, we could classify 32 unique variants as first priority based on ms-scores> 0.1 (meaning > 10% of described patient-variants are missense) and VIPUR scores > 0.7 (Supplementary Table S4). Despite this prioritization, these variants remain VUS and would require additional information (identification in affected individuals or functional testing) to be reportable. However, adding expert knowledge from the literature and structural protein information allowed prioritization and reduction of the number of variants to a manageable amount, a step that will be required for future functional testing.

## Discussion

In this study, we analyzed WES data for 118 ciliopathy genes in ~400 healthy individuals to investigate the carrier frequency for ciliopathies and to identify current challenges in the implementation of NGS-based carrier screening (NGS-CS). Depending on the criteria applied to determine pathogenicity of variants, we found carrier frequencies ranging from 11%, when considering only previously reported certainly pathogenic variants, to 20%, when also including novel LOF alleles. In addition, we found 50% of this healthy population to carry at least one very rare “strong” VUS and identified between 1% (only pathogenic) and 5% (including VUS) of couples at potential risk of having a child affected with a ciliopathy. Our findings are consistent with previous reports on NGS-CS [4, 36, 37]. With more genes analyzed, the proportion of individuals carrying *P/LP* variants increases, as shown by Porter et al. [38]. Beyond the number of genes analyzed, the main factor influencing the carrier frequency is the choice of criteria for interpretation of variant pathogenicity. Indeed, a major challenge lies in the application of the ACMG/AMP variant classification criteria in healthy individuals, in whom phenotypic match, a second allele in trans and/or familial segregation cannot provide support for pathogenicity. Our work shows that the vast majority of missense variants can still not be confidently classified and that NGS-CS will detect a “strong” VUS in 50% of healthy individuals and 4% of couples when analyzing 118 genes. While most providers will follow the ESHG recommendations [9] and not report VUS for carrier screening, not all VUS are equal and different laboratories/providers may interpret the evidence from the literature differently, as also illustrated in the conflicting interpretations in ClinVar or in the comparison between ClinVar and HGMD. For example, a rare missense variant previously reported as pathogenic by a reputed source, because it was found in one patient from a large cohort in trans with a LOF variant in a gene matching the phenotype, may be judged reportable by some providers (based on criteria PM2, PM3, PP2 and PP4 in the reported case) but not by others (single description in the literature and no criteria PM3 and PP4 applicable in the healthy individual screened). The weight given to previous classifications therefore remains an individual decision and no clear consensus exists at the current time for variant classification in the specific context of NGS-CS.

In addition to the question of which variants to report (or not), an additional question lies in the decision of the genes to include in the screening. This selection currently varies between laboratories and countries [39], which often offer various options for the referring provider or couple to choose from. The recently released ACMG guidelines suggest offering NGS-CS to all couples, for genes with carrier frequencies >1/200 based on GnomAD allele frequencies of *P/LP* variants [3]. Our work shows that the true carrier frequency for many rare disorders remains unclear, as illustrated by the discrepancy between observed carrier frequencies and estimated disease prevalence. We show that the carrier frequency even for LOF variants in established ciliopathy disease genes (e.g. *ALMS1*), is higher than expected based on disease prevalence (carrier frequency of ~1/79 for a disease prevalence of ~1/100’000-1’000’000). Similar observations have already been described for other rare disorders [40–43]. Moreover, in the recent ACMG carrier screening guidelines some genes (i.e. *DYNC2H1*) are listed with much higher carrier frequencies (1/50) than expected based on prevalence of the associated disorder (short-rib-polydactyly/Jeune syndrome, prevalence ~1/100’000). Moreover, carrier frequencies based on GnomAD do not necessarily correspond to the epidemiology of the disorders: *BBS2* for example has a higher carrier frequency than *BBS1* in GnomAD [3], while the latter is much more commonly mutated in patients with the corresponding ciliopathy Bardet-Biedl syndrome [44]. The observed high carrier frequencies, even when focusing only on clearly established disease genes and LOF alleles, suggest either that the true disease prevalence is substantially underestimated and/or that some (combinations of) variants may be incompatible with life. Alternatively, these findings could suggest that not all predicted LOF variants are disease-causing, as has also been previously described [45]. The consequences for carrier screening would be important, since even strict observance of ACMG/AMP criteria could yield “false positive” results if a presumed LOF variant does not cause disease. Taken together, these findings suggest that the proposed carrier frequency cut-off based on GnomAD data for selection of genes to screen by NGS-CS has limitations. This could be improved by expert curation of the genes retained after a first pass selection based on GnomAD. By adding gene- and disorder specific knowledge, experts could assist in curating the recommended gene lists: for JBTS for example, the four genes selected in the current recommendations (*CEP290, CC2D2A, TMEM216* and *AHI1*) could be complemented by adding *C5ORF42/CPLANE1* and *TMEM67* which cause the disease with a similar frequency [25]. Unifying NGS-CS gene panels, as is currently already being attempted [46], would be highly beneficial for patients and providers, in order to avoid arbitrary choices not supported by biological or medical knowledge. Moreover, in concordance with the ACMG guidelines, in the current state of knowledge, residual risk after a negative test result should not be communicated, given the large number of VUS, of which it remains unclear how many represent disease alleles.

In the end, we will likely need reliable functional assays to confidently classify rare variants as pathogenic or benign. It therefore remains essential to prioritize which variants to assess through functional assays available today or in the future. In addition to routinely used automated prediction tools, intimate knowledge of the gene variation patterns in patients and of the biology of the encoded protein can provide helpful additional information. The known variation patterns for each gene in patient cohorts can increase or decrease the likelihood that a novel missense variant causes disease. Integrating knowledge about protein structure, which can be added efficiently using VIPUR [35], is of further assistance in prioritization of variants.

Despite the challenges highlighted by the current study, pan-ethnic NGS-CS can be offered responsibly, keeping in mind the inverse correlation between positive and negative predictive values. A high positive predictive value should be prioritized and only pathogenic variants with sufficient supporting information should be reported, since prenatal diagnosis, pregnancy termination or costly pre-implantation genetic diagnosis based on VUS raise ethical concerns [47]. The high number of VUS, and the uncertainties around true disease prevalence and carrier frequency for most rare disorders, underscore the importance of professional pre- and post-test counseling sessions explaining these limitations.

## Supplementary information

Supplemental material

Supplementary Table S4

Supplementary Table S6

Supplementary Table S7

## Data Availability

The supplementary files (including xls spreadsheets) contain all the relevant information. All genomic sequence variants discussed in this work are available in the main manuscript or supplementary files (Supplementary xls tables).
