# Supplementary material for: Challenges for the implementation of next generation sequencing-based expanded carrier screening: Lessons learned from the ciliopathies
Source: Eur J Hum Genet. 2022 Dec 23;31(8):953–61. doi: 10.1038/s41431-022-01267-8 (PMC10400553; doi:10.1038/s41431-022-01267-8)
Supplement: Supplementary file 1 — Supplemental material [file 41431_2022_1267_MOESM1_ESM.docx]

**Challenges for the implementation of NGS-based expanded carrier screening: lessons learned from the ciliopathies**

**- Supplementary information**

Ella Vintschger^1^, Dennis Kraemer^1^, Pascal Joset^1^, Anselm H. C. Horn^2,3^, Anita Rauch^1,3^, Heinrich Sticht^2^, Ruxandra Bachmann-Gagescu^1,3,4^

1 Institute of Medical Genetics, University of Zurich, 8952 Schlieren, Switzerland

2 Institute of Biochemistry, Friedrich-Alexander-Universität Erlangen-Nürnberg (FAU), 91054 Erlangen, Germany

3 Praeclare Clinical Research Priority Program of the Medical Faculty, University of Zurich, Switzerland

4 Department of Molecular Life Sciences, University of Zurich, 8057 Zurich, Switzerland


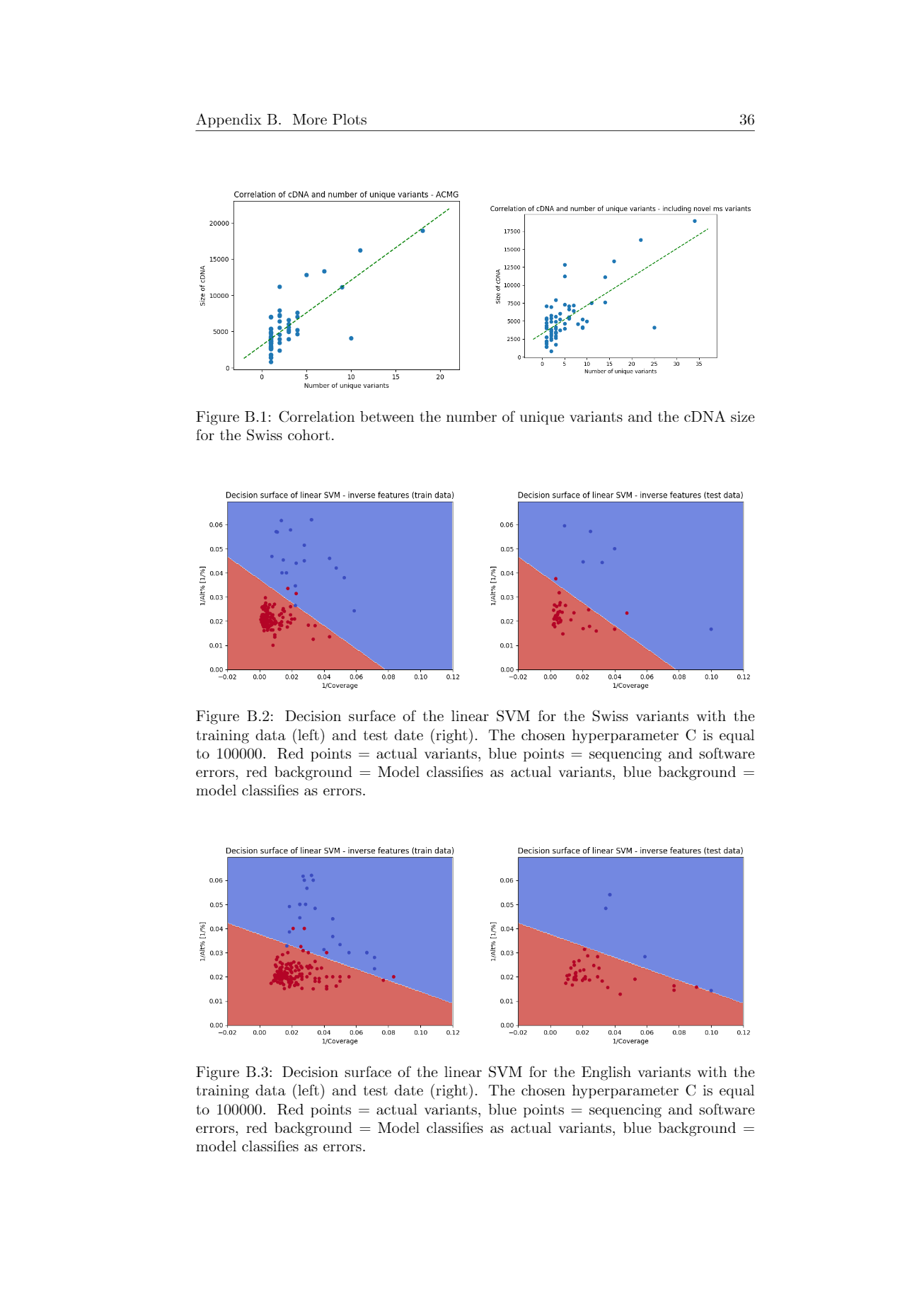


**Supplementary Figure S1: Automated exclusion of false sequencing calls using a linear support vector machine (SVM)**

Graph showing the separation achieved between true calls (red dots) and false calls (blue dots). The red background shows the model’s classification as true calls, while the blue background indicates the model’s prediction of false calls. The SVM was trained and tested on 253 manually labeled variants from 12 individuals (training set 172 variants, test set 81 variants). The model yields a specificity of 0.987 and a sensitivity of 0.947 on the training set (0.971 and 1 on the test set). The decision to rather allow a small number of false negatives (i.e. to exclude a few true calls from the analysis) rather than to include any false positives was taken in order to not overestimate the carrier frequency in this healthy population; in a clinical diagnostic setting, the priorities would obviously be different and the SVM would be trained accordingly.

Base position

**in house cohort**

**UK1958 cohort**

**Supplementary Figure S2: Sequencing coverage of exome data for the *in house* cohort of healthy individuals and for the UK1958 cohort**

Plot showing the coverage distribution for the whole exome sequencing data for the local *in house* cohort (blue) and for the UK1958 cohort (orange). Average coverage was 286.9 reads per basepair for the local cohort and 57.2 reads per basepair for the UK cohort.


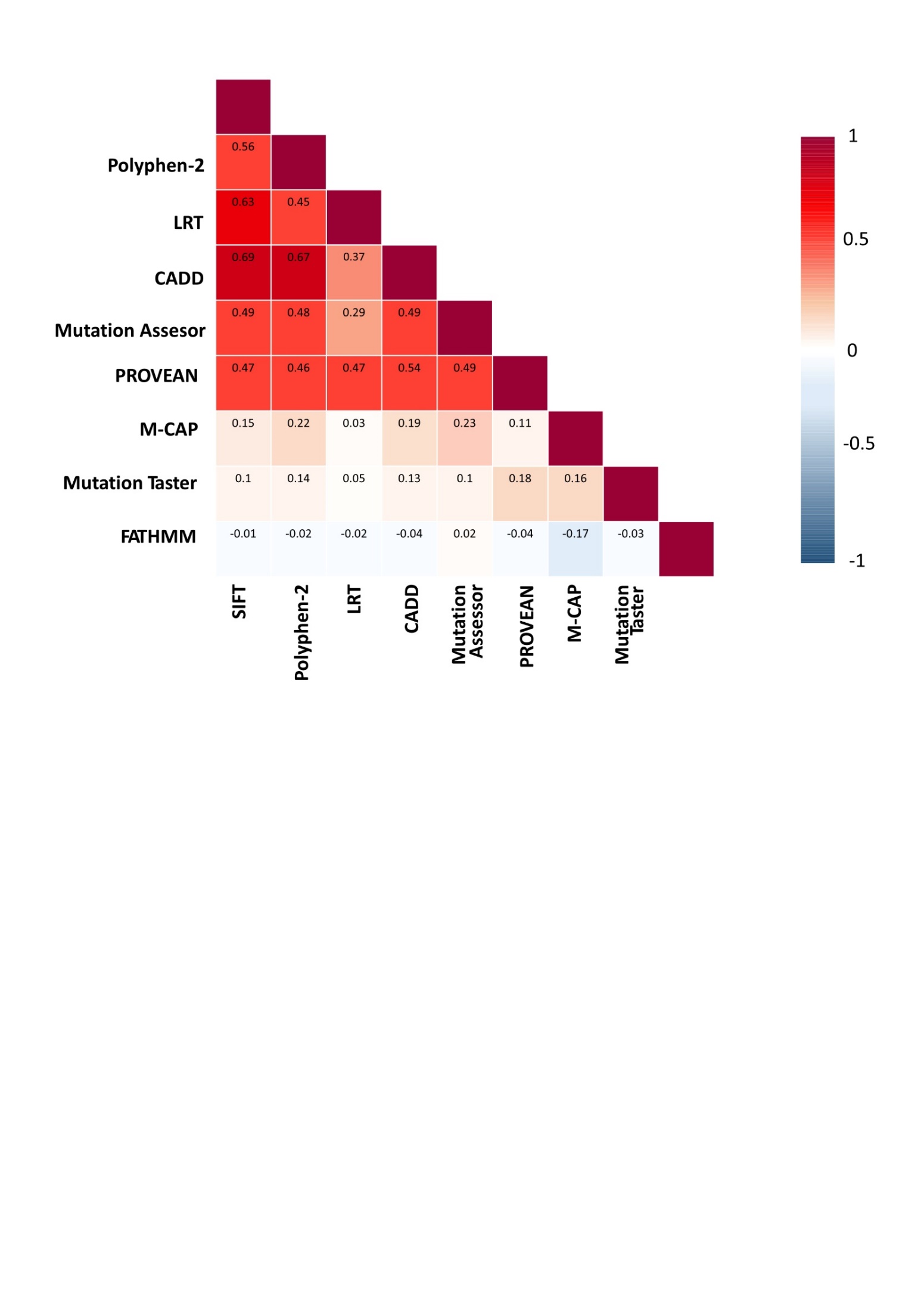


**Supplementary Figure S3: Comparison of pathogenicity classifications between different commonly used prediction tools**

Graphical representation of the Pearson correlation from pairwise comparisons between SIFT-Polyphen2-LRT-MutationTaster-MutationAssessor-FATHMM-Provean-M-CAP and CADD scores using the numerical scores of each prediction tool. Red represents a positive correlation (agreement) and blue a negative correlation (disagreement), darker colors indicate higher values of the correlation coefficient. Note that the pairwise correlations show a moderate to good agreement (score around 0.5) between Polyphen2, LRT, CADD, Mutation Assessor, Provean and SIFT, with the best correlation between Polyphen2 and CADD and between SIFT and CADD.


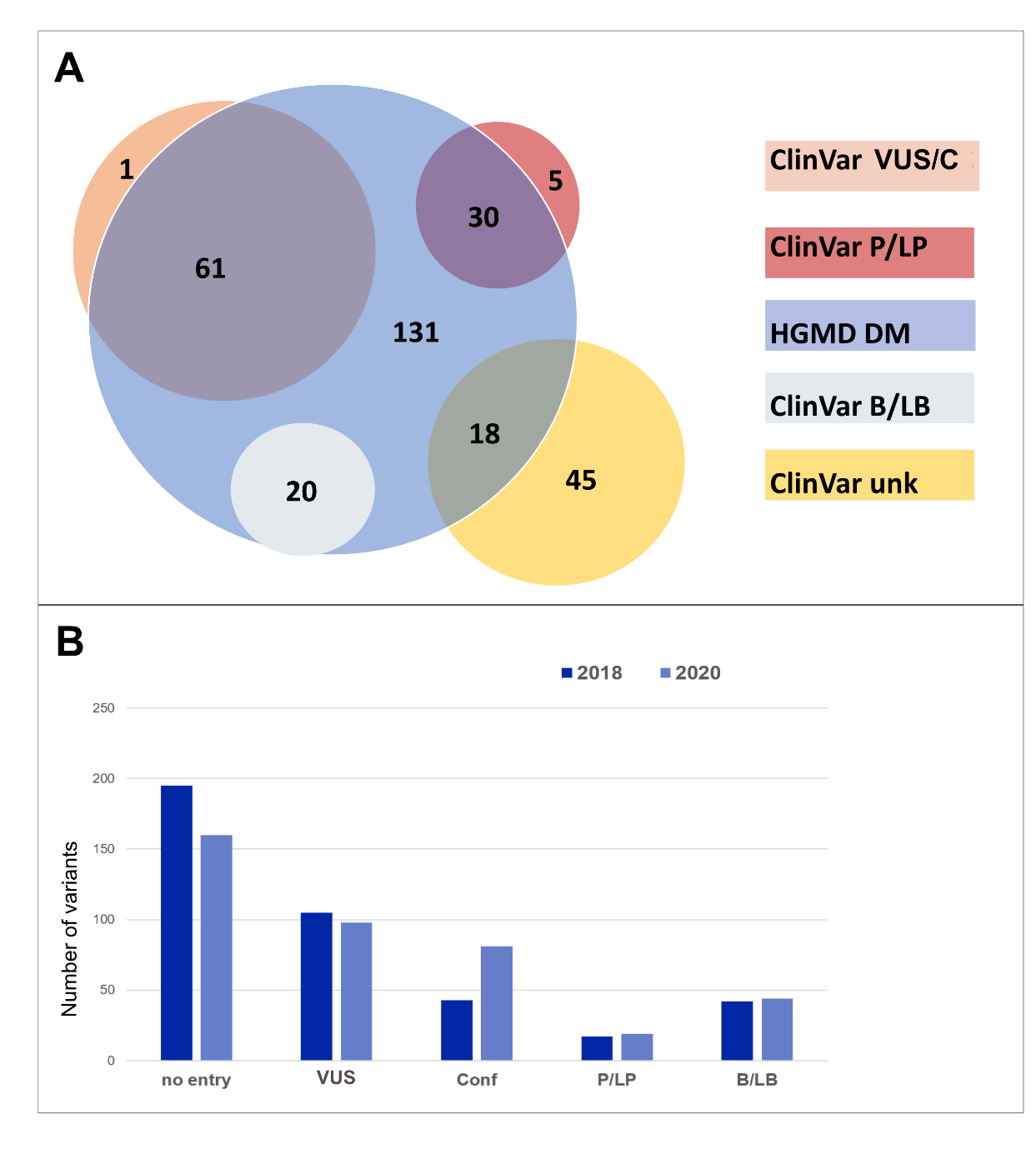


**Supplementary Figure S4: Comparison of variant classification databases and evolution over time**

**(A)** Comparison of ClinVar and HGMD classifications for 182 missense variants identified in our dataset. Venn Diagram showing the intersection of the different classifications between these two databases. Of 131 *DM* HGMD variants (blue circle), only 30 were pathogenic or likely pathogenic (*P/LP*) in ClinVar (red circle), while 61 were classified as variants of unknown significance (VUS) or with conflicting interpretations of pathogenicity (*C*) in ClinVar (pink circle) and 20 variants were benign or likely benign (*B/LB*) in ClinVar (light grey circle). Note that prior to generating this plot, we had already excluded 63 “*DM*-variants” from HGMD due to a MAF >0.4%. **(B)** Changes in the ClinVar classification for 400 missense variants from our dataset between 2018 and 2020. Note that the number of variants without an entry in ClinVar has decreased over these two years, but that this is paralleled by an increase in variants with conflicting interpretations (*Conf*). Variants with definite classification as Benign/Likely benign (*B/LB*) or Pathogenic/likely pathogenic (*P/LP*) have barely increased.

**Supplementary Table S1: Demographic information for the local cohort of healthy individuals**

| **Total number of individuals** |  | | | 395 |
| --- | --- | --- | --- | --- |
| **Average Age (years)** |  | | | 44 |
| **Gender** | Female | | | 206 |
|  | Male | | | 189 |
| **Couples** | Non-consanguineous | | | 156 |
|  | consanguineous | | | 21 |
| **Ancestry** | Caucasian | | | 334 |
|  | Non-Caucasian | |  | 61 |
|  |  | | Asian | 21 |
|  |  | | African | 29 |
|  |  | American | | 5 |
|  |  | Uncertain/unknown | | 6 |

| **Supplementary Table S2: Ciliopathy disorders and genes taken into account in this study** | | | | | | | | | | | | | | | | | | | | | |
| --- | --- | --- | --- | --- | --- | --- | --- | --- | --- | --- | --- | --- | --- | --- | --- | --- | --- | --- | --- | --- | --- |
| **HGNC gene name** | **OMIM gene name** | **other gene name** | **JBTS** | **MKS** | **OFD** | **ACLS** | **HLS** | **BBS** | **Alstrom** | **MKKS** | **JATD** | **SRP** | **CED** | **EVC** | **NPHP** | **SLS** | **Usher** | **LCA** | **RP** | **CRD** |  |
| ***ADGRV1*** | ***USH2C*** | ***GPR98*** |  |  |  |  |  |  |  |  |  |  |  |  |  |  | X |  |  |  |  |
| ***AHI1*** | ***JBTS3*** |  | X |  |  |  |  |  |  |  |  |  |  |  |  |  |  |  |  |  |  |
| ***ALMS1*** | ***ALMS1*** | ***KIAA0328*** |  |  |  |  |  |  | X |  |  |  |  |  |  |  |  |  |  |  |  |
| ***ANKS6*** | ***NPHP16*** |  |  |  |  |  |  |  |  |  |  |  |  |  | X |  |  |  |  |  |  |
| ***ARL13B*** | ***JBTS8*** | ***ARL2L1*** | X |  |  |  |  |  |  |  |  |  |  |  |  |  |  |  |  |  |  |
| ***ARL3*** | ***JBTS35, RP83*** | ***ARFL3*** | X |  |  |  |  |  |  |  |  |  |  |  |  |  |  |  | X |  |  |
| ***ARMC9*** | ***JBTS30*** | ***KIAA1868*** | X |  |  |  |  |  |  |  |  |  |  |  |  |  |  |  |  |  |  |
| ***B9D1*** | ***JBTS27, ?MKS9*** | ***MKSR1*** | X | X |  |  |  |  |  |  |  |  |  |  |  |  |  |  |  |  |  |
| ***B9D2*** | ***JBTS34, ?MKS10*** | ***MKSR2*** | X | X |  |  |  |  |  |  |  |  |  |  |  |  |  |  |  |  |  |
| ***BBIP1*** | ***?BBS18*** |  |  |  |  |  |  | X |  |  |  |  |  |  |  |  |  |  |  |  |  |
| ***BBS1*** | ***BBS1*** |  |  |  |  |  |  | X |  |  |  |  |  |  |  |  |  |  | X |  |  |
| ***BBS10*** | ***BBS10*** | ***C12ORF58*** |  |  |  |  |  | X |  |  |  |  |  |  |  |  |  |  |  |  |  |
| ***BBS12*** | ***BBS12*** | ***C4ORF24*** |  |  |  |  |  | X |  |  |  |  |  |  |  |  |  |  |  |  |  |
| ***BBS2*** | ***BBS2, RP74*** |  |  |  |  |  |  | X |  |  |  |  |  |  |  |  |  |  | X |  |  |
| ***ARL6*** | ***BBS3, RP55*** |  |  |  |  |  |  | X |  |  |  |  |  |  |  |  |  |  | X |  |  |
| ***BBS4*** | ***BBS4*** |  |  |  |  |  |  | X |  |  |  |  |  |  |  |  |  |  |  |  |  |
| ***BBS5*** | ***BBS5*** |  |  |  |  |  |  | X |  |  |  |  |  |  |  |  |  |  |  |  |  |
| ***MKKS*** | ***BBS6*** |  |  |  |  |  |  | X |  | X |  |  |  |  |  |  |  |  |  |  |  |
| ***BBS7*** | ***BBS7*** |  |  |  |  |  |  | X |  |  |  |  |  |  |  |  |  |  |  |  |  |
| ***TTC8*** | ***BBS8, ?RP51*** |  |  |  |  |  |  | X |  |  |  |  |  |  |  |  |  |  | X |  |  |
| ***BBS9*** | ***BBS9*** | ***PTHB1*** |  |  |  |  |  | X |  |  |  |  |  |  |  |  |  |  | X |  |  |
| ***C2CD3*** | ***OFDIV*** |  | X |  | X |  |  |  |  |  |  |  |  |  |  |  |  |  |  |  |  |
| ***PCARE*** | ***RP54*** | ***C2ORF71*** |  |  |  |  |  |  |  |  |  |  |  |  |  |  |  |  | X |  |  |
| ***C8orf37*** | ***BBS21, RP64, CRD16*** |  |  |  |  |  |  | X |  |  |  |  |  |  |  |  |  |  | X | X |  |
| ***CC2D2A*** | ***JBTS9, MKS6*** | ***KIAA1345*** | X | X |  |  |  |  |  |  |  |  |  |  |  |  |  |  |  |  |  |
| ***CDH23*** | ***USH1D*** |  |  |  |  |  |  |  |  |  |  |  |  |  |  |  | X |  |  |  |  |
| ***CEP104*** | ***JBTS25*** | ***KIAA0562*** | X |  |  |  |  |  |  |  |  |  |  |  |  |  |  |  |  |  |  |
| ***CEP120*** | ***JBTS31, SRTD13*** |  | X |  |  |  |  |  |  |  |  | X |  |  |  |  |  |  |  |  |  |
| ***CEP164*** | ***NPHP15*** | ***KIAA1052*** |  |  |  |  |  |  |  |  |  |  |  |  | X |  |  |  |  |  |  |
| ***CEP41*** | ***JBTS15*** | ***TSGA14*** | X |  |  |  |  |  |  |  |  |  |  |  |  |  |  |  |  |  |  |
| ***CEP290*** | ***NPHP6,MKS4 JBTS5,LCA10?BBS14,*** |  | X | X |  |  |  | X |  |  |  |  |  |  | X | X |  | X |  |  |  |
| ***CEP78*** | ***CRDHL1*** |  |  |  |  |  |  |  |  |  |  |  |  |  |  |  |  |  |  | X |  |
| ***CEP83*** | ***NPHP18*** | ***CCDC41*** |  |  |  |  |  |  |  |  |  |  |  |  | X |  |  |  |  |  |  |
| ***CIB2*** | ***USH1J*** |  |  |  |  |  |  |  |  |  |  |  |  |  |  |  | X |  |  |  |  |
| ***CPLANE1*** | ***JBTS17, OFDVI*** | ***C5ORF42*** | X |  | X | X |  |  |  |  |  |  |  |  |  |  |  |  |  |  |  |
| **HGNC gene** | **OMIM gene** | **other de-nomination** | **JBTS** | **MKS** | **OFD** | **ACLS** | **HLS** | **BBS** | **Alstrom** | **MKKS** | **JATD** | **SRP** | **CED** | **EVC** | **NPHP** | **SLS** | **Usher** | **LCA** | **RP** | **CRD** |  |
| ***CSPP1*** | ***JBTS21*** | ***CSPP*** | X |  |  |  |  |  |  |  |  |  |  |  |  |  |  |  |  |  |  |
| ***DCDC2*** | ***NPHP19*** |  |  |  |  |  |  |  |  |  |  |  |  |  | X |  |  |  |  |  |  |
| ***DDX59*** | ***OFDV*** |  |  |  | X |  |  |  |  |  |  |  |  |  |  |  |  |  |  |  |  |
| ***DYNC2H1*** | ***SRTD3*** |  |  |  |  |  |  |  |  |  | X | X |  |  |  |  |  |  |  |  |  |
| ***DYNC2LI1*** | ***SRTD15*** | ***D2LIC*** |  |  |  |  |  |  |  |  |  | X |  |  |  |  |  |  |  |  |  |
| ***EVC*** | ***EVC*** |  |  |  |  |  |  |  |  |  |  |  |  | X |  |  |  |  |  |  |  |
| ***EVC2*** | ***EVC2*** |  |  |  |  |  |  |  |  |  |  |  |  | X |  |  |  |  |  |  |  |
| ***EXOC8*** | ***EXOC8*** | ***SEC84*** | X |  |  |  |  |  |  |  |  |  |  |  |  |  |  |  |  |  |  |
| ***FAM161A*** | ***RP28*** |  |  |  |  |  |  |  |  |  |  |  |  |  |  |  |  |  | X |  |  |
| ***GLIS2*** | ***NPHP7*** |  |  |  |  |  |  |  |  |  |  |  |  |  | X |  |  |  |  |  |  |
| ***HYLS1*** | ***HYLS1*** |  |  |  |  |  | X |  |  |  |  |  |  |  |  |  |  |  |  |  |  |
| ***IFT122*** | ***CED1*** |  |  |  |  |  |  |  |  |  |  | X | X |  |  |  |  |  |  |  |  |
| ***IFT140*** | ***SRTD9, RP80*** |  |  |  |  |  |  |  |  |  |  | X |  |  |  |  |  | X | X |  |  |
| ***IFT172*** | ***SRTD10, RP71*** | ***KIAA1179*** | X |  |  |  |  |  |  |  |  | X |  |  | X |  |  |  | X |  |  |
| ***IFT27*** | ***?BBS19*** |  |  |  |  |  |  | X |  |  |  |  |  |  |  |  |  |  |  |  |  |
| ***IFT43*** | ***SRTD18, ?CED3, ?RP81*** |  |  |  |  |  |  |  |  |  |  | X | X |  |  |  |  |  | X |  |  |
| ***IFT52*** | ***SRTD16*** |  |  |  |  |  |  |  |  |  |  | X |  |  |  |  |  |  |  |  |  |
| ***IFT57*** | ***?OFDXVIII*** | ***HIPPI*** |  |  | X |  |  |  |  |  |  |  |  |  |  |  |  |  |  |  |  |
| ***IFT74*** | ***?BBBS20*** | ***CCDC2*** |  |  |  |  |  | X |  |  |  |  |  |  |  |  |  |  |  |  |  |
| ***IFT80*** | ***SRTD2*** | ***WDR56*** |  |  |  |  |  |  |  |  | X | X |  |  |  |  |  |  |  |  |  |
| ***IFT81*** | ***SRTD19*** |  |  |  |  |  |  |  |  |  |  | X |  |  |  |  |  |  |  |  |  |
| ***INPP5E*** | ***JBTS1, MORM*** |  | X |  |  |  |  |  |  |  |  |  |  |  |  |  |  |  |  |  |  |
| ***INTU*** | ***?SRTD20, ?OFDXVII*** |  |  |  |  |  |  |  |  |  |  | X |  |  |  |  |  |  |  |  |  |
| ***KATNIP*** | ***JBTS26*** | ***KIAA0556*** | X |  |  |  |  |  |  |  |  |  |  |  |  |  |  |  |  |  |  |
| ***KIAA0586*** | ***JBTS23, SRTD14*** |  | X |  |  |  |  |  |  |  |  | X |  |  |  |  |  |  |  |  |  |
| ***KIAA0753*** | ***?OFDXV*** |  |  |  | X |  |  |  |  |  |  |  |  |  |  |  |  |  |  |  |  |
| ***KIF14*** | ***?MKS12*** | ***KIAA0042*** |  | X |  |  |  |  |  |  |  |  |  |  |  |  |  |  |  |  |  |
| ***KIF7*** | ***ACLS,?HLS2, JBTS12*** |  | X |  |  | X | X |  |  |  |  |  |  |  |  |  |  |  |  |  |  |
| ***LCA5*** | ***LCA5*** | ***C6ORF152*** |  |  |  |  |  |  |  |  |  |  |  |  |  |  |  | X |  |  |  |
| ***LZTFL1*** |  |  |  |  |  |  |  | X |  |  |  |  |  |  |  |  |  |  |  |  |  |
| ***MAK*** |  |  |  |  |  |  |  |  |  |  |  |  |  |  |  |  |  |  | X |  |  |
| ***MKS1*** | ***MKS1*** |  | X | X |  |  |  | X |  |  |  |  |  |  |  |  |  |  |  |  |  |
| ***MYO7A*** |  |  |  |  |  |  |  |  |  |  |  |  |  |  |  |  | X |  |  |  |  |
| ***NEK1*** |  |  |  |  |  |  |  |  |  |  |  | X |  |  |  |  |  |  |  |  |  |
| ***NPHP1*** |  |  | X |  |  |  |  |  |  |  |  |  |  |  | X | X |  |  |  |  |  |
| ***NPHP11*** |  |  |  |  |  |  |  |  |  |  |  |  |  |  | X |  |  |  |  |  |  |
| ***INVS*** | ***NPHP2*** |  |  |  |  |  |  |  |  |  |  |  |  |  | X |  |  |  |  |  |  |
| ***NPHP3*** |  |  | X | X |  |  |  |  |  |  |  |  |  |  | X |  |  |  |  |  |  |
| **HGNC gene** | **OMIM gene** | **other de-nomination** | **JBTS** | **MKS** | **OFD** | **ACLS** | **HLS** | **BBS** | **Alstrom** | **MKKS** | **JATD** | **SRP** | **CED** | **EVC** | **NPHP** | **SLS** | **Usher** | **LCA** | **RP** | **CRD** |  |
| ***NPHP4*** |  |  |  |  |  |  |  |  |  |  |  |  |  |  | X | X |  |  |  |  |  |
| ***IQCB1*** | ***NPHP5*** |  |  |  |  |  |  |  |  |  |  |  |  |  | X | X |  | X | X |  |  |
| ***GLIS2*** | ***NPHP7*** |  |  |  |  |  |  |  |  |  |  |  |  |  | X |  |  |  |  |  |  |
| ***NEK8*** | ***NPHP9*** |  |  |  |  |  |  |  |  |  |  |  |  |  | X |  |  |  |  |  |  |
| ***PCDH15*** |  |  |  |  |  |  |  |  |  |  |  |  |  |  |  |  | X |  |  |  |  |
| ***PDE6D*** |  |  | X |  |  |  |  |  |  |  |  |  |  |  |  |  |  |  |  |  |  |
| ***PDZD7*** |  |  |  |  |  |  |  |  |  |  |  |  |  |  |  |  | X |  |  |  |  |
| ***PIBF1*** |  |  | X |  |  |  |  |  |  |  |  |  |  |  |  |  |  |  |  |  |  |
| ***PKHD1*** |  |  |  |  |  |  |  |  |  |  |  |  |  |  |  |  |  |  |  |  |  |
| ***POC1B*** |  |  | X |  |  |  |  |  |  |  |  |  |  |  |  |  |  |  |  | X |  |
| ***RAB28*** |  |  |  |  |  |  |  |  |  |  |  |  |  |  |  |  |  |  |  | X |  |
| ***RP1*** |  |  |  |  |  |  |  |  |  |  |  |  |  |  |  |  |  |  | X |  |  |
| ***RPGRIP1*** |  |  |  |  |  |  |  |  |  |  |  |  |  |  |  |  |  | X | X | X |  |
| ***RPGRIP1L*** | ***NPHP8*** |  | X | X |  |  |  |  |  |  |  |  |  |  | X |  |  |  |  |  |  |
| ***SDCCAG8*** | ***NPHP10*** |  |  |  |  |  |  | X |  |  |  |  |  |  | X | X |  |  |  |  |  |
| ***SCLT1*** |  |  |  |  | X |  |  |  |  |  |  |  |  |  |  |  |  |  |  |  |  |
| ***SEPT7*** |  |  |  |  |  |  |  | X |  |  |  |  |  |  |  |  |  |  |  |  |  |
| ***SPATA7*** |  |  |  |  |  |  |  |  |  |  |  |  |  |  |  |  |  | X | X |  |  |
| ***SUFU*** |  |  | X |  |  |  |  |  |  |  |  |  |  |  |  |  |  |  |  |  |  |
| ***TBC1D32*** |  |  |  |  | X |  |  |  |  |  |  |  |  |  |  |  |  |  |  |  |  |
| ***TCTEX1D2*** |  |  |  |  |  |  |  |  |  |  |  | X |  |  |  |  |  |  |  |  |  |
| ***TCTN1*** |  |  | X |  |  |  |  |  |  |  |  |  |  |  |  |  |  |  |  |  |  |
| ***TCTN2*** |  |  | X | X |  |  |  |  |  |  |  |  |  |  |  |  |  |  |  |  |  |
| ***TCTN3*** |  | ***C10ORF61*** | X |  | X |  |  |  |  |  |  |  |  |  |  |  |  |  |  |  |  |
| ***TMEM107*** |  |  | X | X | X |  |  |  |  |  |  |  |  |  |  |  |  |  |  |  |  |
| ***TMEM138*** |  |  | X |  |  |  |  |  |  |  |  |  |  |  |  |  |  |  |  |  |  |
| ***TMEM216*** | ***MKS2*** |  | X | X |  |  |  |  |  |  |  |  |  |  |  |  |  |  |  |  |  |
| ***TMEM231*** |  |  | X | X |  |  |  |  |  |  |  |  |  |  |  |  |  |  |  |  |  |
| ***TMEM237*** |  |  | X |  |  |  |  |  |  |  |  |  |  |  |  |  |  |  |  |  |  |
| ***TMEM67*** | ***MKS3*** |  | X | X |  |  |  |  |  |  |  |  |  |  | X |  |  |  |  |  |  |
| ***TOPORS*** |  |  |  |  |  |  |  |  |  |  |  |  |  |  |  |  |  |  | X |  |  |
| ***TRAF3IP1*** |  |  |  |  |  |  |  |  |  |  |  |  |  |  |  | X |  |  |  |  |  |
| ***TRIM32*** | ***BBS11*** | ***HT2A*** |  |  |  |  |  | X |  |  |  |  |  |  |  |  |  |  |  |  |  |
| ***TTC21B*** |  | ***THM1*** | X |  |  |  |  |  |  |  | X | X |  |  | X |  |  |  |  |  |  |
| ***TTLL5*** |  |  |  |  |  |  |  |  |  |  |  |  |  |  |  |  |  |  |  | X |  |
| ***TULP1*** |  |  |  |  |  |  |  |  |  |  |  |  |  |  |  |  |  | X | X |  |  |
| ***USH1C*** |  |  |  |  |  |  |  |  |  |  |  |  |  |  |  |  | X |  |  |  |  |
| ***USH1E*** |  |  |  |  |  |  |  |  |  |  |  |  |  |  |  |  | X |  |  |  |  |
| ***USH1G*** |  |  |  |  |  |  |  |  |  |  |  |  |  |  |  |  | X |  |  |  |  |
| ***USH2A*** |  |  |  |  |  |  |  |  |  |  |  |  |  |  |  |  | X |  | X |  |  |
| ***WDPCP*** |  |  |  |  | X |  |  | X |  |  |  |  |  |  |  |  |  |  |  |  |  |
| ***WDR19*** |  | ***IFT144*** |  |  |  |  |  |  |  |  | X | X |  |  | X | X |  |  |  |  |  |
| ***WDR34*** |  |  |  |  |  |  |  |  |  |  |  | X |  |  |  |  |  |  |  |  |  |
| ***WDR35*** |  |  |  |  |  |  |  |  |  |  |  | X |  |  |  |  |  |  |  |  |  |
| ***WDR60*** |  |  |  |  |  |  |  |  |  |  |  | X |  |  |  |  |  |  |  |  |  |
|  |  | **Total** | **39** | **13** | **6** | **2** | **2** | **23** | **1** | **1** | **4** | **19** | **2** | **2** | **22** | **7** | **10** | **10** | **34** | **13** |  |
|  |  |  | **JBTS** | **MKS** | **OFD** | **ACLS** | **HLS** | **BBS** | **Alstrom** | **MKKS** | **JATD** | **SRP** | **CED** | **EVC** | **NPHP** | **SLS** | **Usher** | **LCA** | **RP** | **CRD** |  |

**Disorders:**

JBTS – Joubert syndrome

MKS – Meckel syndrome

OFD – oro-facio-digital syndrome

ACLS – acro-callosal syndrome

HLS – hydrolethalus syndrome

BBS – Bardet-Biedl syndrome

Alstrom – Alstrom syndrome

MKKS – McKusick-Kaufmann syndrome

JATD – Jeune asphyxiating thoracic dystrophy

SRP – short rib polydactyly

CED – cranioectodermal dysplasia

EVC – Ellis van Creveld syndrome

NPHP - nephronophthisis

SLS – Senior Loken syndrome

Usher – Usher syndrome

LCA – Leber congenital amaurosis

RP – retinitis pigmentosa

CRD – cone-rod dystrophy

X-liked genes including OFD1, RP2 and RPGR were not included.

**Supplementary Table S3: Comparison of results between *in house* cohort and the UK1958 birth cohort**

|  | **Local in house cohort (n=395 individuals)** | | | | **UK1958 Cohort (n=999 individuals)** | | | |
| --- | --- | --- | --- | --- | --- | --- | --- | --- |
| **Coverage** | **286.9** | | | | **57.2** | | | |
| **Quality score (NextGen softgenetics; range)** | **10.7-27.7** | | | | **7.3-19.3** | | | |
| **Total n high confidence variants** | **3702** | | | | **7377** | | | |
| **N variants per individual (range)** | **1-24** | | | | **1-19** | | | |
| **Average N variants per individual** | **9.32** | | | | **7.38** | | | |
|  | **reportable variants (pathogenic ACMG/AMP criteria)** | | **“strong” VUS ***  **or LP by ACMG/AMP criteria** | | **reportable variants (pathogenic ACMG/AMP criteria)** | | **“strong” VUS ***  **or LP by ACMG/AMP criteria** | |
| **Total variants** | **86** | | **265** | | **190** | | **566** | |
|  | ***P* ClinVar / *DM* HGMD** | **48** | *LP* ClinVar | 5 | ***P* ClinVar/HGMD** | **84** | *LP* ClinVar or *DM* HGMD | **147** |
|  | LOF / truncating | 26 | *DM* HGMD | 42 | LOF/truncating | **46** | LOF/truncating | **0** |
|  | ms/nc/in-frame | 22 | ms/nc/in-frame (VUS or absent ClinVar / DM? or absent HGMD) | 218 | ms/nc/in-frame | **38** | ms/nc/in-frame (VUS or absent ClinVar, DM? or absent HGMD) | **419** |
|  | **Novel LOF/ truncating variants** | **38** |  |  | **Novel LOF/ truncating variants** | **106** |  |  |
| **Individuals carrying variants** (**%**) | **80 (20%)** | | **197 (50%)** | | **154 (15%)** | | **449 (45%)** | |
| **N variants/individual (range)** | 0-2 | | 0-5 | | 0-2 | | 0-5 | |
| **Avg variants/individual** | 0.23 | | 0.66 | | 0.19 | | 0.57 | |
| **Couples at risk (%)** | 2 /177 (1%) | | 7/177 (4%) | | NA | | NA | |

Number of variants identified in our local cohort and in the UK1958 birth cohort. Reportable variants are light grey and VUS in dark grey. The slightly lower numbers in the UK1958 cohort might be due to the lower sequencing coverage leading to proportionately fewer high quality calls retained. *DM* disease associated, *HGMD* human gene mutation database, *LOF* loss-of-function, *LP* likely pathogenic, *ms* missense, *N* number, *NA* not applicable, *nc* non coding, *P* pathogenic, VUS Variant of uncertain significance.

**Supplementary Table S4: Detailed xls spreadsheets of all variants**

Tab “pathogenic variants”: contains all details for the variants judged pathogenic and reportable; column names are self-explanatory. The same variant may appear more than once if it recurs in more than one individual in the cohort.

Tab “novel LOF variants”: contains predicted LOF variants (nonsense, frameshift, canonical splice site) that had no entry in ClinVar or HGMD.

Tab “LP variants”: variants present in ClinVar with classification of likely pathogenic or predicted loss-of-function classified as VUS in ClinVar.

Tab “VUS 2018_2020”: contains variants that are considered “strong” VUS and thus not reportable for carrier screening: very rare and predicted to be deleterious by multiple *in silico* prediction tools. Many variants in this category were classified as DM in HGMD, but evidence was not deemed sufficient to consider them as pathogenic. The columns ClinVar Significance 2018_07 and 2020_04 show the changes in interpretation over a span of 2 years.

Tab “all variants pooled”: includes all variants retained from our dataset either as pathogenic, novel LOF, LP or VUS (all previous tabs pooled) – sorted by patient ID to highlight how some individuals have more than one variant (can be only VUS or pathogenic and VUS).

Tab “VUS prioritized VIPUR_ms”: contains the 32 VUS that can be prioritized (for example for functional assays) based on a missense score (ms score) > 0.1 and a VIPUR score > 0.7. All columns in red represent the parameters kept for making the decision of prioritizing these variants. The yellow/orange color coding for the ms score reflects the strength of the information available from the literature based on the number of entries in varsome (yellow = <10 entries, orange = between 10 and 20 entries).

**Supplementary Table S5: Couples with a possible reproductive risk for ciliopathies**

| **Family ID** | **gene** | **aa change** | **N prediction softwares** | **M_CAP** | **CADD** | **GnomAD MAF** | **Homo/Hemizygous in GnomAD** | **HGMD** | **Clinvar** | **VIPUR** | **Manual re-classification** | **Disorder** |
| --- | --- | --- | --- | --- | --- | --- | --- | --- | --- | --- | --- | --- |
| **235 (cons)** | ***KIAA0586*** | p.K25Rfs | NA | NA |  |  |  | NA | NA | NA | **LP** |  |
|  | ***DYNC2H1*** | p.R2532RW | 5/7 | D | 25.9 | 0 | 0 | **DM** | P | D | **P** | Jeune |
| **130 (cons)** | ***KIAA0556*** | splice | NA | NA |  | 0.0004 | 0 | NA |  | NA | **LP** | JBTS |
| **138** | ***USH2A*** | p.S4275ST | 1/7 | D | 0.006 | 0.0002 | 0 | **DM** | VUS | NA | VUS | USH |
|  |  | p.E3939EK | 2/7 | D | 21.7 | 0.0004 | 1 | **DM** | VUS | neut | VUS |  |
| **144** | ***USH2A*** | p.C759FC | 8/7 | D | 33 | 0.0002 | 0 | **DM** | P/LP | D | **LP** | USH |
|  |  | p.T2197IT | 3/7 | D | 27.3 | 0 | 0 | **DM** | VUS | NA | VUS |  |
| **101 (cons)** | ***IFT81*** | A.348TA | 5/7 | D | 32 | 0 | 0 | NA | NA | neut | VUS | SRP |
| **133** | ***USH2A*** | p.3232PQ | 5/7 | D | 20.7 | 0 | 0 | NA | NA | NA | VUS | USH |
|  |  | p.3258RQ | 3/7 | D | 35 | 0 | 0 | NA | NA | NA | VUS |  |
| **135** | ***CEP290*** | p.K1220NK | 5/7 | D | 24.1 | 0.0002 | 0 | NA | VUS | neut | VUS | JBTS, |
|  |  | p.N1094NK | 5/7 | D | 25.7 | 0 | 0 | NA | NA | neut | VUS | NPHP, LCA |
| **66** | ***DYNC2H1*** | p.M1Rfs | NA | NA |  | 0 | 0 | NA | NA | NA | **LP** | Jeune |
|  |  | p.C1772YC | 4/7 | D | 28.5 | 0 | 0 | NA | NA | NA | VUS |  |
| **108** | ***DYNC2H1*** | p.G4035GC | 4/7 | D | 32 | 0 | 0 | NA | VUS | D | VUS | Jeune |
|  |  | p.I2819IM | 4/7 | D | 23.6 | 0 | 0 | **DM** | VUS | neut | VUS |  |

Orange cells show couples where both partners harbor variants that are deemed reportable in the same ciliopathy gene; both these couples are consanguineous (*cons*). In 7 couples, both partners harbor variants that we judge as VUS and that would not be reportable; however, all of these variants are very rare and are predicted to be deleterious by multiple prediction tools and several of them have been classified as DM in HGMD.

The disorders associated with the respective genes are listed in the last column: *Jeune* Jeune asphyxiating thoracic dystrophy, *JBTS* Joubert syndrome, *RP* Retinitis Pigmentosa, *USH* Usher syndrome, *SRP* Short-Rib-Polydactyly, *LCA* Leber Congenital Amaurosis, *NPHP* Nephronophthisis.

Abbreviations: *B* Benign, *C* Conflicting, *D* Deleterious, *DM* disease-causing mutation, *LP* likely pathogenic, *NA* not available, *neut* neutral, *P* pathogenic, *T* Tolerated, *VUS* Variant of Uncertain Significance

**Supplementary Table S6 (xls. document): recurrent missense and truncating variants in our dataset (*in house cohort*)**

**Supplementary Table S7 (xls. document): Calculation of the missense score (“ms-score”) based on *Varsome* clinical statistics table**

**Supplementary methods:**

**Manual pre-processing of variants from WES raw data results:**

We manually removed redundant calls for each frameshift variant to retain a single description for each frameshift variant. Known calling artefacts were also manually removed, as well as 6 variants with insufficient quality lacking crucial parameters such as %Alt or /coverage which were required for the next step (SVM, see below). We also excluded calls that concerned genes present on the other DNA strand opposite the ciliopathy gene of interest.

**Training of the linear support vector machine (SVM) to exclude false calls:**

The SVM was trained and tested using previously manually labeled variants from 12 individuals (253 variants). The SVM is a method to separate with the largest possible margin two groups by a function, which in our case is a linear function. The training set consisted of 70% of the labeled data (172 variants in the local cohort), whereas the test set contained 30% (81 variants). The sets were separated by a cut-off, so some individuals had variants in both sets. The hinge-loss was chosen as the minimization target. By using GridSearch, the best value for the penalty parameter C was found to be 100000, indicating a strong penalty for wrongly classified points. To account for the difference in number between actual variants and sequencing/software errors in the mutation-reports, the two classes were weighted accordingly. Coverage and the percentage of the alternate (variant) nucleotide at the variant position (Alt%) showed the best correlation with the label, which was further enhanced by using the respectively inverse value. The model yields a specificity of 0.987 and a sensitivity of 0.947 on the training data and 0.971and 1 on the testing data, respectively. For the UK1958 birth cohort the SVM had to be trained separately, due to the large difference in sequencing coverage between the two cohorts. In the UK1958 cohort data, 18 individuals (a total of 203 variants) were manually labeled. The training set consisted of approximately 80% of the variants. The same model approach was used as with the *in house* data: the penalty parameter C was set to 100000 and the model consisted of a linear SVM with the inverted values of the coverage and Alt%. The model yields a specificity of 0.964 and a sensitivity of 0.957 on the training data and 1 and 1 on the testing data, respectively.

**Variant filtering**

Criteria applied for variant pathogenicity determination relied on following standard databases/algorithms (“prediction tools”):

| **Acronym** | **Full name** | **link** | **Variants retained** | **Date accessed** |
| --- | --- | --- | --- | --- |
| **GnomAD** | Genome Aggregation Database | <https://gnomad.broadinstitute.org/> | MAF<0.1% | 20.05.2019 |
| **ClinVar** | ClinVar | <https://www.ncbi.nlm.nih.gov/clinvar/> | P (LP) | 01.07.2018, 20.04.2020 |
| **HGMD** | Human Gene Mutation Database |  | DM (DM?) | 20.05.2019 |
| **SIFT** | Sorting Intolerant from Tolerant | <https://sift.bii.a-star.edu.sg/> | D | 20.05.2019 |
| **PolyPhen 2 HVAR** | Polymorphism Phenotyping version 2 HumVar | <http://genetics.bwh.harvard.edu/pph2/> | D | 20.05.2019 |
| **LRT** | Likelihood Ratio Test |  | L | 20.05.2019 |
| **Mutation Taster** | Mutation Taster | <https://www.mutationtaster.org/> | D,A | 20.05.2019 |
| **Mutation Assessor** | Mutation Assessor | <http://mutationassessor.org/r3/> | M.H | 20.05.2019 |
| **FATHMM** | Functional Analysis through Hidden Markov Models | <http://fathmm.biocompute.org.uk/> | D | 20.05.2019 |
| **PROVEAN** | Protein Variation Effect Analyzer | <http://provean.jcvi.org/index.php> | D | 20.05.2019 |
| **M-CAP** | Mendelian Clinically Applicable Pathogenicity | <http://bejerano.stanford.edu/mcap/> | D | 20.05.2019 |
| **CADD** | Combined Annotation Dependent Depletion | <https://cadd.gs.washington.edu/> | >20 | 20.05.2019 |
